# Are Routine Radiographs Needed the Day After Open Reduction and Internal Fixation Surgery for Distal Radius and Ankle Fractures: Study Protocol for a Prospective, Open Label, Randomized Controlled Trial

**DOI:** 10.2196/resprot.7698

**Published:** 2017-08-16

**Authors:** Florian Oehme, Annika Rühle, Julia Mühlhäusser, Lana Fourie, Björn-Christian Link, Reto Babst, Frank JP Beeres

**Affiliations:** ^1^ Lucerne Cantonal Hospital Surgery Department Lucerne Switzerland; ^2^ Lucerne Cantonal Hospital Orthopaedic and Trauma Surgery Lucerne Switzerland

**Keywords:** wrist fracture, distal radius fracture, ankle fracture, postoperative radiograph, functional outcome, cost reduction, radiation exposure

## Abstract

**Background:**

Distal radius and ankle fractures are one of the most common operatively treated fractures. To date, there is no consensus concerning the need for a standard postoperative radiograph. This leads to undesirable practice variations. A standardized radiograph in the department of radiology would theoretically be more reproducible and operator independent than an intraoperatively obtained fluoroscopic image. However, if adequate intraoperative radiographs have been obtained, it is questionable if these postoperative radiographs are necessary and will lead to changes in the treatment strategy. If standard postoperative radiographs are no longer required, this would lead to a reduction in radiation exposure and health care costs. The hypothesis is that routine standardized postoperative radiographs do not influence the quality of care for patients operated on for either a distal radius or an ankle fracture if adequate intraoperative standardized radiographs have been obtained.

**Objective:**

The primary aim of this study is to evaluate if there is a need for routine postoperative radiographs after an osteosynthesis of a distal radius or ankle fracture.

**Methods:**

In a prospective, randomized controlled, open label trial based on a noninferiority design, we will enroll 332 patients. Patients will be randomized either in the control or the intervention group. The control group will be treated according to our current, standard protocol in which all patients receive a standard anterior-posterior and lateral radiograph on the first postoperative day. Patients randomized to the intervention group will be treated without a standard postoperative radiograph. All patients (N=332) will have a routine clinical and radiographic control after 6 weeks in the outpatient clinic. Primary outcome is a change in treatment plan, defined as either additional imaging or a reoperation based on the postoperative imaging. Secondary outcome measures include a 36-Item Short Form Survey, Patient-Rated Wrist Hand Evaluation, Foot and Ankle Outcome Score, Visual Analogue Scale, and the range of motion. Those questionnaires will be filled out at the 6-week outpatient control.

**Results:**

The trial was started in August 2016, and 104 patients have been enrolled up to this point.

**Conclusions:**

Our findings will be reported in peer-reviewed publications and may lead to a strong reduction in radiation exposure and health care costs. A preliminary, conservative estimation suggests a yearly cost saving of CHF 1.3 million in Switzerland.

## Introduction

In a society that is increasingly demanding about the functional outcome after treatment of fractures, the indications for operative treatment have increased over the past decade. Wrist fractures specifically are more often being treated operatively. In addition, due to an increase in the number of patients with obesity, the numbers of patients treated for ankle fractures are also increasing [1,2].

Many hospitals still use routine postoperative radiographs, despite the fact that intraoperative images have been obtained for reduction and implant control. In addition, these images are stored and available in the Picture Archiving and Communication System (PACS). These postoperative radiographs lead to a significant increase in radiation exposure and costs. It remains questionable if these standard postoperative radiographs are justified since the quality of intraoperative C-arm images has improved over the last decade.

### Rationale

Intraoperative radiograph documentation, if standardized and adequately performed, has the potential to assess the quality of reduction and fixation [3,4]. Consequently, the additional value of routine postoperative radiographs should be questioned [5].

Given the trend towards cost-effective medicine [6] and keeping cumulative radiation exposure in mind, it is not surprising that these standardized postoperative radiographs are under debate [8]. Despite the above, routine postoperative radiographs are still being performed in many hospitals as a standard of care. Many surgeons and radiologists argue that radiographs obtained in the department of radiology are more standardized and less biased. We therefore evaluated the frequency of changes in treatment plan due to standardized postoperative radiographs in a retrospective trial. Changes in treatment plan were defined as a deviation from the standard postoperative protocol. This included additional imaging or a revision operation.

We found in 7.2% of patients a change in the treatment plan following the evaluation of the standardized postoperative radiograph for patients operated on for distal radius or ankle fractures in 2014. These numbers are high in percentage and suggest that standardized postoperative radiographs do add to the quality of care for the patient.

On the other hand, this retrospective study had several methodological drawbacks, especially as the intraoperative radiographs performed were not standardized. Therefore, we decided to evaluate the need for postoperative radiographs using a prospective randomized control trial. We would expect a significant reduction in radiation exposure and costs of around CHF 1.3 million (estimated for employed patients in Switzerland in 2015 [9]).

### Hypothesis

Our hypothesis is that routine standardized postoperative radiographs do not influence the quality of care for patients operated for a distal radius or ankle fracture if adequate intraoperative standardized radiographs have been obtained.

This is the first prospective randomized trial evaluating the additional value of postoperative routine radiographs in operative fracture care of distal radius and ankle fractures.

Human research ethics approval has been obtained from the Ethikkomission Nordwest- und Zentralschweiz (EKNZ). The commission accepted this trial on April 4, 2016 (EKNZ BASEC 2016-00114).

## Methods

Based on a noninferiority design, we planned a prospective, randomized controlled, open label trial. Multimedia Appendix 1 shows the study protocol in accordance with the Standard Protocol Items: Recommendations for Interventional Trials (SPIRIT) checklist [10].

### Study Population

The study includes patients operated for a distal radius or ankle fracture at a Level 1 trauma center in Switzerland. Patients presenting with these fractures at the emergency department will be eligible for study inclusion if they fulfill all of the following criteria: indication for an operation of a distal radius fracture, according to the Arbeitsgemeinschaft für Osteosynthesefragen (AO) Classification types 23-A-C [4], or an ankle (according to the AO Classification Types 44-A-C) fracture, 18 years of age or older, sufficient understanding and writing of German language, and signed informed consent. The informed consent forms will be stored by the study nurse during the trial.

Patients are excluded if they meet at least one of the following exclusion criteria: not willing/able to sign the informed consent, indication for postoperative computed tomography (CT), pathological fractures, open fractures (>grade I according to Anderson and Gustilo [11]), patients not able to attend the 6 weeks outpatient control, or missed intraoperative standardized radiographs.

### Preliminary

Essential pre-study preparations are needed to ensure standardization of intraoperative C-arm handling, limb positioning, and reproducibility of the collected data.

#### Defining Period

Standard intraoperative radiographs for patients operated for radius fractures are defined as:

1. anterior-posterior and posterior-anterior radiograph of the wrist with a free visualization of the distal radio-ulnar space (defined as no overlap of distal radius and distal ulnar)
2. 20° radial tilted lateral view in which the os pisiform needs to have an overlap with the scaphoid and the ventral cortex of the lunate
3. tangential view [12,13]

**Figure 1:**
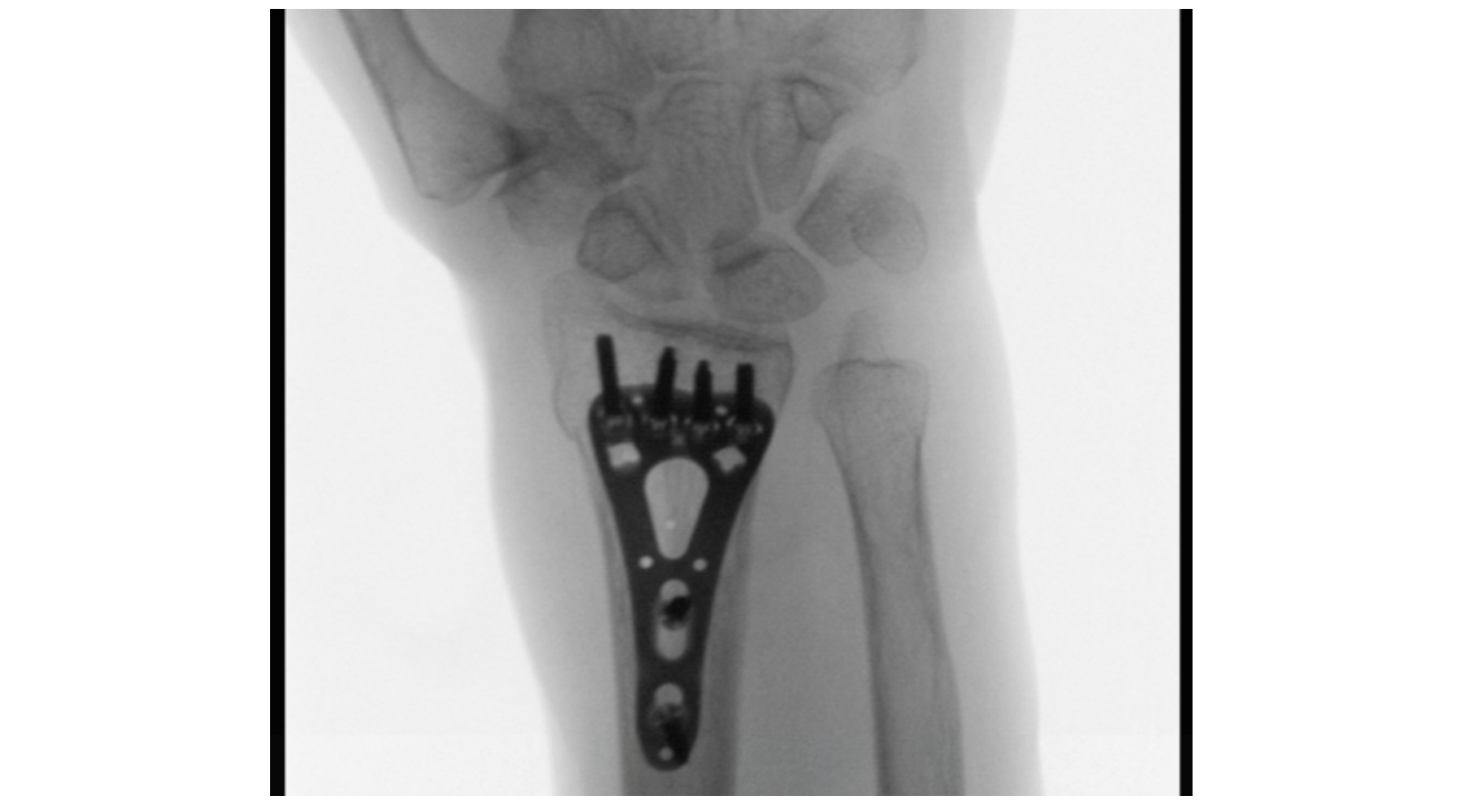
Intraoperative C-arm controlled radiograph (anterior-posterior) in supination with cleared radio-ulnar space.

**Figure 2:**
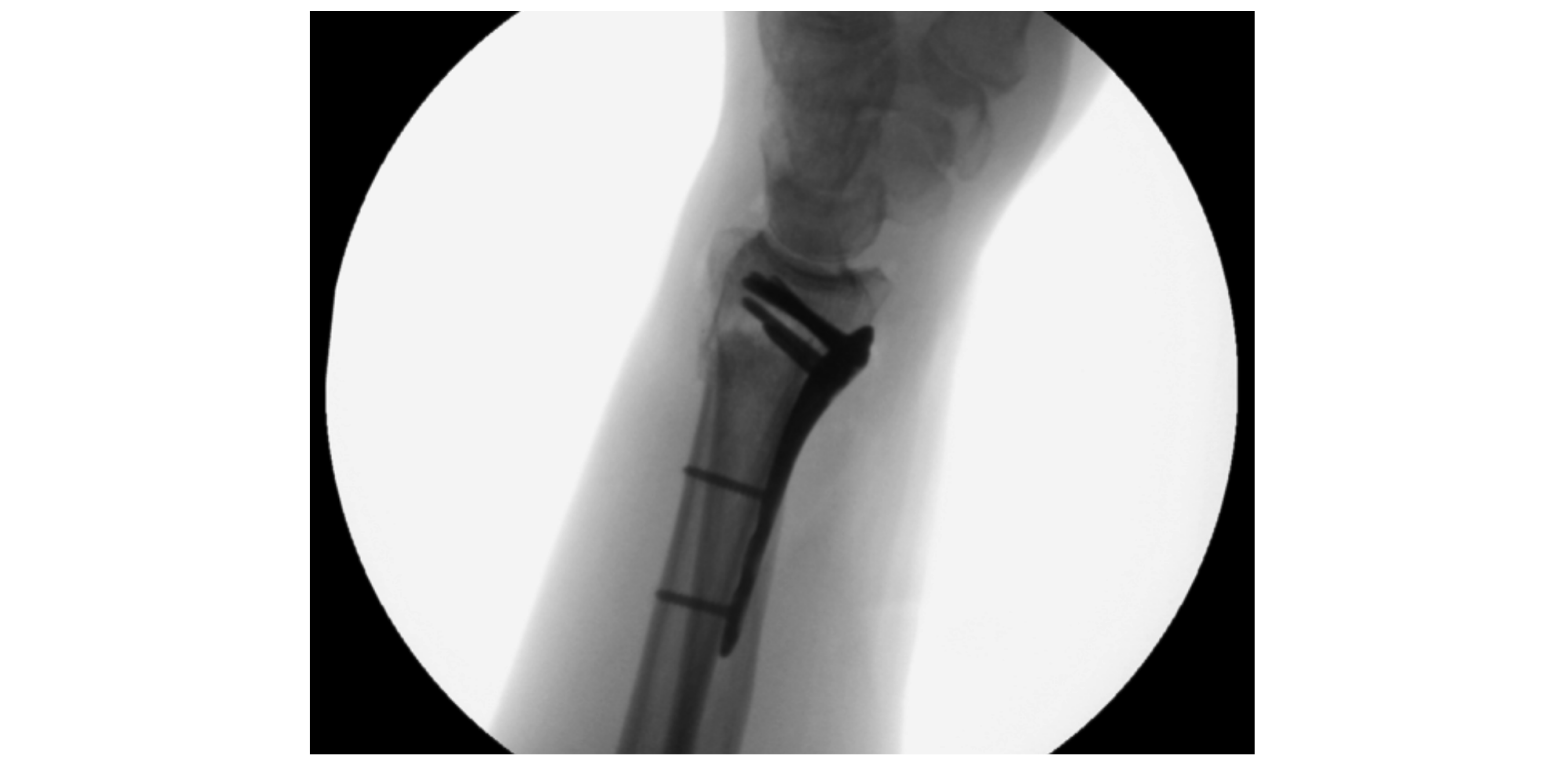
Lateral view with free os pisiforme on the volar level and intra-articular view.

**Figure 3:**
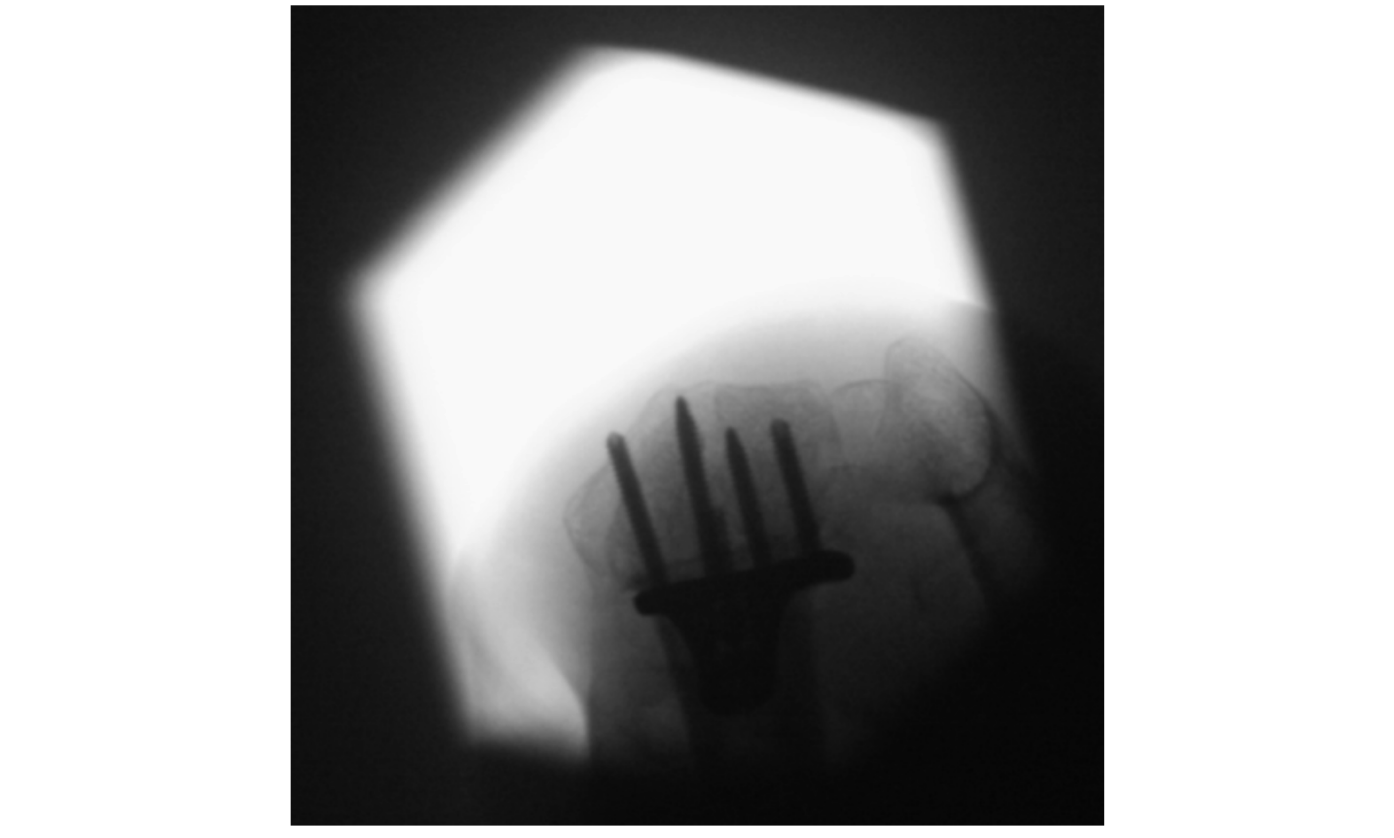
Intraoperative tangential view performed to evaluate the length of the screws.

Sufficient standard intraoperative radiographs are shown in Figures 1,2, and 3.

Standard intraoperative radiographs for patients operated for ankle fractures are defined as:

1. anterior-posterior view (mortise view) with cleared space between trochlea tali and the tibia/fibula (achieved by inward rotation of 20°)
2. true lateral view with an overlap of the medial and lateral domes of the talus and the fibula forming the posterior one-third of the tibia

**Figure 4:**
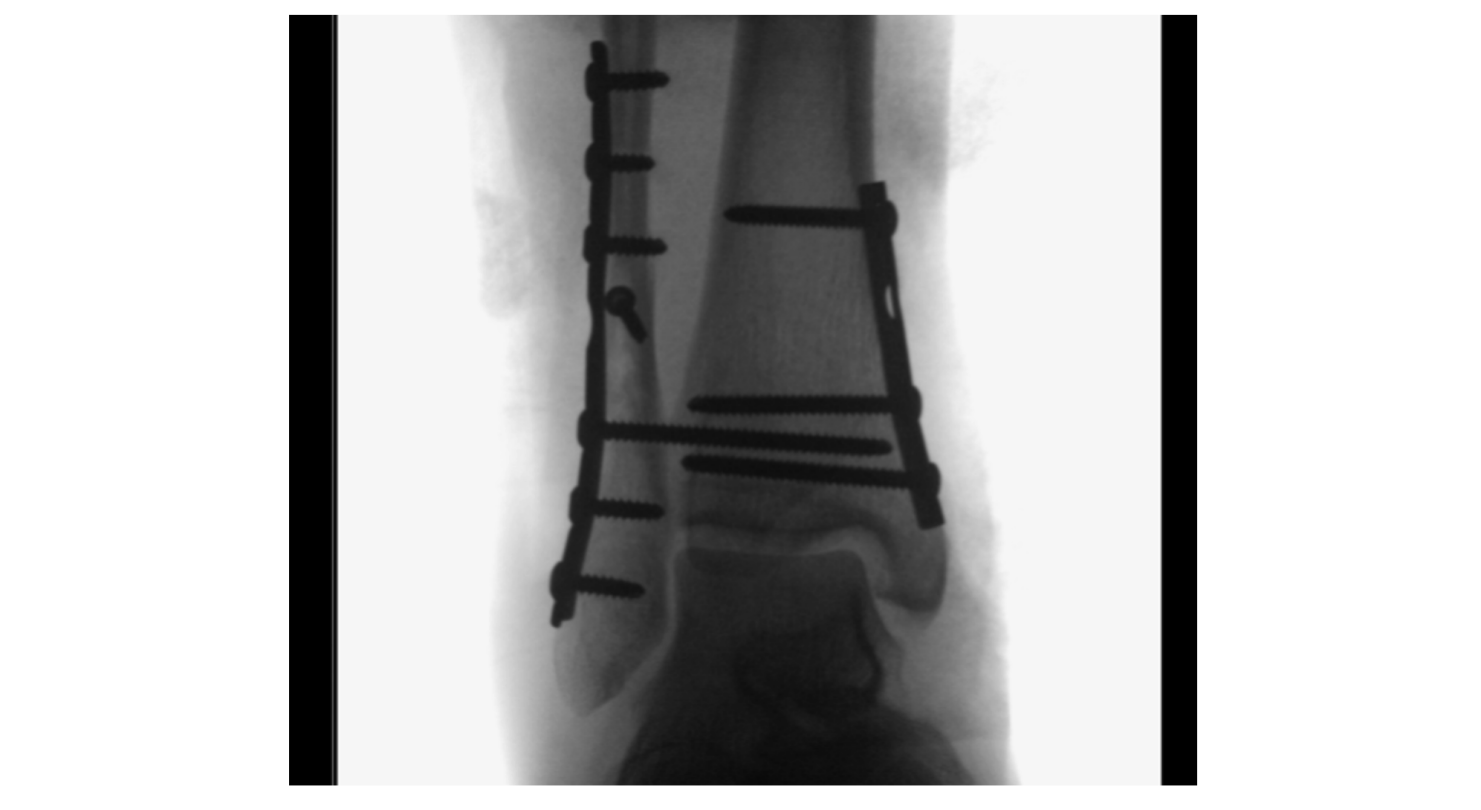
Intraoperative C-arm control: the ankle is shown in anterior-posterior view, free mortise view is achieved with 90° dorsal extension and 20° rotation inside.

**Figure 5:**
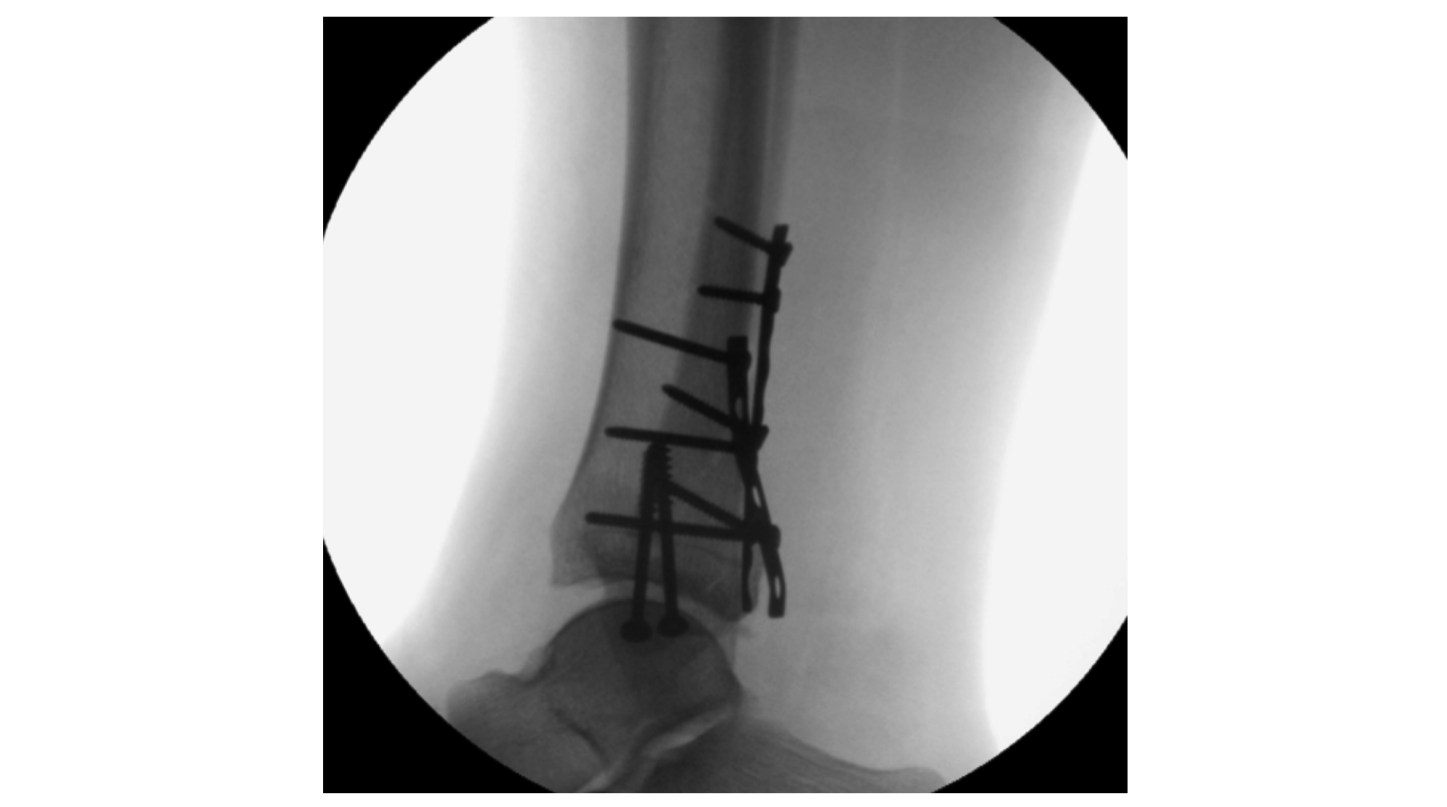
Intraoperative C-arm control: the ankle is shown in a lateral view, to show the trochlea tali for intra-articular view; the fibula forms the posterior one-third of the tibia.

Sufficient standard intraoperative radiographs are shown in Figures 4 and 5.

#### Implementation Period

An educational period teaching standard intraoperative radiographs will be initiated before the inclusion period starts. All trauma surgeons treating the above mentioned fractures will participate in a special lecture about standardization of intraoperative radiographs. Additionally, a tailored personal instruction lecture by the senior surgeon will be initiated.

In a last educational step, personal hand-outs, providing information about appropriate standardization, are given to each trauma surgeon as well as a special lecture involving the residents and interns who participate in orthopedic trauma surgery. They are informed about the necessity of a signed informed consent as the surgeon at the emergency department is responsible for the inclusion.

#### Inclusion Period

After sufficient information is provided to all trauma surgeons, inclusion starts. Intraoperative radiographs are obtained by the ZIEHM SOLO (Ziehm Imaging GmbH). These radiographs are saved separately in our PACS. The next day, these radiographs are evaluated by the senior surgeon of the department of orthopedics and trauma surgery and a senior radiologist. If the quality of the intraoperative radiographs is standardized and good, the patient remains in the study arm to which they were assigned.

### Randomization

Prior to the operation, the patients are randomized to the control or the intervention group. The randomization will be carried out using a special in-hospital randomization tool. Thereafter, the patient will be included in one of the following two groups.

#### Control Group (Group I)

Patients belonging to the control group will be treated according to our current protocol, including a standardized postoperative radiograph. Regular postoperative radiographs are performed in the department of radiology and contain an anterior-posterior and lateral tilted view of the wrist. In patients with an ankle fracture, a lateral view and a mortise view are obtained. Generally, patients operated on for a distal radius fracture will have a functional after treatment and will be restricted in weight bearing for the first 6 weeks postoperative. Operatively treated ankle fractures will generally receive a functional after treatment with weight bearing restricted to 15 kg for 6 weeks. All patients will have a clinical and radiological outpatient control after 6 weeks.

#### Intervention Group (Group II)

Patients belonging to the intervention group will have a modified postoperative protocol. After interdisciplinary consensus (between the head of the orthopedics department and trauma surgery and radiology) that the quality of the intraoperative radiographs are performed in a standardized fashion with good quality and that the reposition is within the predefined standards, patients will be mobilized according to our routine postoperative protocol and will be reviewed in our outpatient department after 6 weeks (clinically and radiologically).

If the quality of the intraoperative radiographs or the reduction is not acceptable, additional imaging (plain radiographs or CT) or a revision operation is performed.

### Outcome

As we present a noninferiority approach, the purpose of this analysis is to show comparable results in the intervention group in terms of functional outcome and safety for the patients.

#### Primary Outcome

We defined the primary outcome as any re-intervention or necessity for additional imaging due to insufficient intraoperative imaging or achieved reduction.

#### Secondary Outcome

Secondary outcome is measured using different specific functional outcome scores as well as nonspecific outcome tools.

### Specific Tools

The following tools used correspond to the specific fracture treated.

#### Patient-Rated Wrist Hand Evaluation

Patients operated for distal radius fractures will fill out the Patient-Rated Wrist Hand Evaluation. This 15-item questionnaire includes both a section concerning functional outcome as well as a section that analyzes the associated pain. In both sections, the items (range from 0 reflecting no pain/disability to 100) are added up and the sum is divided by two.

#### Range of Motion

Additionally the range of motion (ROM) will be measured during the outpatient clinical visits. The ROM of the wrist will be tested by measuring the palmar flexion, dorsal extension, pronation, supination, and radial and ulnar deviation.

#### Foot and Ankle Outcome Score

Patients operated for ankle fractures have to complete the Foot and Ankle Outcome Score (FAOS Score). This score was developed to assess the patient’s opinion about nonspecific ankle problems. The FAOS consists of 5 subscales that investigate pain, function in daily living, function in sport and recreation, foot and ankle-related quality of life, and other symptoms. Standardized answer options are given (% Likert boxes) and each question gets a score from 0-4. A normalized score (100 indicating no symptoms and 0 indicating extreme symptoms) is calculated for each subscale.

### Nonspecific Tool

Health status measurement will be performed using the Short-Form 36-Item Health Survey (SF-36) in all patients. The SF-36 is a patient-reported survey of the patient’s health [7]. The questionnaire includes eight different sections (ie, vitality, physical functioning, bodily pain, general health perceptions, physical role functioning, emotional role functioning, social role functioning, mental health). Each section has a scoring range between 0 and 100. The score is proportional to the outcome with the best possible result of 100.

The visual analogue scale is used on all our patients at the 6-week control. This scale ranges from 0-10 where 0 equals no pain and 10 the worst imaginable pain.

Additionally, a medical questionnaire answered by the trauma surgeon in the outpatient clinic investigates whether any signs of a complex regional pain syndrome or complications are present.

### Statistical Analysis

Statistical analysis will be carried out using SPSS, IBM Version 21. In order to answer the primary research questions, the number of re-interventions will be compared between both groups. Additionally, we will use regression *models* to try to identify risk factors for these re-interventions such as timing and duration of surgery and the experience of the surgeon.

In order to address the secondary research questions, the evaluated specific and nonspecific outcome scores will be compared between the two groups using means and standard deviation.

### Sample Size Calculation

We determined the sample size for both the radius and ankle fracture group after a retrospective evaluation concerning the rate of postoperative treatment plan changes. We found a rate of 7.2% changes in treatment plan after obtaining standard postoperative radiographs in 2014. Based on these findings and using a power of 0.80 and alpha failure of .05, we performed a sample size calculation.

Based on a noninferiority approach, we calculated 158 patients needed in each treatment arm. Corresponding to the standard protocol following operative fracture care, we have a standard first outpatient control after 6 weeks. Based on a separate evaluation, concerning the frequency of patients being lost to follow-up, 4.6% of our patients in 2014 did not obtain their 6-week control. Therefore we added 5% loss of follow-up to our power calculation, resulting in 158 x 1.05=166 patients in each treatment arm.

### Ethical Considerations

The study design is in accordance with the Declaration of Helsinki [14] and with Swiss laws like the human research act (HFG) and the human research regulation (HFV).

This study was approved by the medical research ethics committee of Basel. The EKNZ accepted this trial on April 4, 2016 (EKNZ BASEC 2016-00114).

All forms handed out to the patient and the information that will be obtained using the above mentioned questionnaires are approved by the EKNZ. Essential changes in the course of the trial will be reported immediately and need to be approved by the ethics committee. Information as well as results will not be presented to the EKNZ on a regular basis. However, for data verification, authorized representatives of the project manager or the ethics committee do have access to the medical data relevant to the project, including the medical history of participants at any time.

Serious adverse events must be reported immediately and if potential life-threatening complications occur, the trial will be stopped until safety is proven by the ethics committee. Patients participating in this clinical trial are covered by a special hospital insurance. This insurance is free for patients and covers any damage or potential damage as well as death caused by the study.

## Results

The trial was started in August 2016 and 104 patients have been enrolled up to this point. See Multimedia Appendix 2 for the schedule of enrollment.

## Discussion

### Principal Considerations

To date, standard postoperative radiographs are often obtained after operatively treated fractures despite the fact that the value of these standardized postoperative radiographs remains under debate. Considering a trend towards cost-effective medicine and keeping the cumulative radiation exposure in mind, this discussion is not surprising. Initial studies in 1996 showed a lack of additional information gained by the postoperative radiograph [15,16] compared to obtained intraoperative radiographs.

A prospective randomized trial is needed to investigate whether a change to a protocol without standard postoperative radiographs is justifiable or not. Therefore, our study is important to proving that standard postoperative radiographs are not needed and that the outcome of patients without a standard postoperative radiograph is comparable with those treated according to the current protocol (ie, postoperative radiographs are routinely performed).

### Strengths and Limitations

This randomized trial will provide prospective data for a common health care problem and the appropriate aftercare. Due to its randomized design, this trial will provide high-quality evidence with clearly predefined objective results.

Outpatient control, for all patients 6 weeks postoperatively, performed by a specialized (orthopedic) trauma surgeon ensures comparable and objective analysis in terms of early surgical outcome.

As a secondary outcome measurement, functional analysis using validated questionnaires will provide additional information. If this trial demonstrates comparability in terms of quality in aftercare between both groups, this could be the basis for a change in standard postoperative process with savings in radiation exposure and health care costs.

Limitations of this study are the single-center design and the lack of blinding during the study period.

### Conclusion

With this prospective randomized trial, we will provide data on the necessity for postoperative standardized radiographs after open reduction and internal fixation of distal radius and ankle fractures. This could lead to a change in the standard postoperative protocol after operative fracture care and will help reduce radiation exposure during the postoperative course.

## Appendix

Multimedia Appendix 1 SPIRIT checklist.

Multimedia Appendix 2 Time schedule of enrollment and assessment for patients participating in this trial.

## Abbreviations

AO: Arbeitsgemeinschaft für Osteosynthesefragen
CT: computed tomography
EKNZ: Ethikkomission Nordwest- und Zentralschweiz
FAOS: Foot and Ankle Outcome Score
PACS: Picture Archiving and Communication System
ROM: range of motion
SF-36: Short-Form 36-Item Health Survey
