# Supplementary material for: Are Routine Radiographs Needed the Day After Open Reduction and Internal Fixation Surgery for Distal Radius and Ankle Fractures: Study Protocol for a Prospective, Open Label, Randomized Controlled Trial
Source: JMIR Res Protoc. 2017 Aug 16;6(8):e159. doi: 10.2196/resprot.7698 (PMC5577453; doi:10.2196/resprot.7698)
Supplement: Multimedia Appendix 1 [file resprot_v6i8e159_app1.pdf]

SPIRIT

STANDARD PROTOCOL ITEMS: RECOMMENDATIONS FOR INTERVENTIONAL TRIALS

SPIRIT 2013 Checklist: Recommended items to address in a clinical trial protocol and related documents*

Section/item	Item No	Description	Addressed on page number
**Administrative information**			
Title	1	Descriptive title identifying the study design, population, interventions, and, if applicable, trial acronym	Page 1, title
Trial registration	2a	Trial identifier and registry name. If not yet registered, name of intended registry	page 1
	2b	All items from the World Health Organization Trial Registration Data Set	page 1
Protocol version	3	Date and version identifier	page 1
Funding	4	Sources and types of financial, material, and other support	Page 6, Acknowledgement
Roles and responsibilities	5a	Names, affiliations, and roles of protocol contributors	page6
	5b	Name and contact information for the trial sponsor	page 6
	5c	Role of study sponsor and funders, if any, in study design; collection, management, analysis, and interpretation of data; writing of the report; and the decision to submit the report for publication, including whether they will have ultimate authority over any of these activities	page 6

	5d	Composition, roles, and responsibilities of the coordinating centre, steering committee, endpoint adjudication committee, data management team, and other individuals or groups overseeing the trial, if applicable (see Item 21a for data monitoring committee)	page 6

Introduction

Background and rationale	6a	Description of research question and justification for undertaking the trial, including summary of relevant studies (published and unpublished) examining benefits and harms for each intervention	page 2, rationale
	6b	Explanation for choice of comparators	page 2, rationale
Objectives	7	Specific objectives or hypotheses	page 2, hypothesis
Trial design	8	Description of trial design including type of trial (eg, parallel group, crossover, factorial, single group), allocation ratio, and framework (eg, superiority, equivalence, noninferiority, exploratory)	page 2, methods/design

Methods: Participants, interventions, and outcomes

Study setting	9	Description of study settings (eg, community clinic, academic hospital) and list of countries where data will be collected. Reference to where list of study sites can be obtained	page 2, study population
Eligibility criteria	10	Inclusion and exclusion criteria for participants. If applicable, eligibility criteria for study centres and individuals who will perform the interventions (eg, surgeons, psychotherapists)	page 2, study population
Interventions	11a	Interventions for each group with sufficient detail to allow replication, including how and when they will be administered	page 2/3, study population
	11b	Criteria for discontinuing or modifying allocated interventions for a given trial participant (eg, drug dose change in response to harms, participant request, or improving/worsening disease)	page 5, ethical considerations
	11c	Strategies to improve adherence to intervention protocols, and any procedures for monitoring adherence (eg, drug tablet return, laboratory tests)	not applicable

	11d	Relevant concomitant care and interventions that are permitted or prohibited during the trial	page 3, defining period
Outcomes	12	Primary, secondary, and other outcomes, including the specific measurement variable (eg, systolic blood pressure), analysis metric (eg, change from baseline, final value, time to event), method of aggregation (eg, median, proportion), and time point for each outcome. Explanation of the clinical relevance of chosen efficacy and harm outcomes is strongly recommended	page 4, outcome
Participant timeline	13	Time schedule of enrolment, interventions (including any run-ins and washouts), assessments, and visits for participants. A schematic diagram is highly recommended (see Figure)	page 4, intervention/control group
Sample size	14	Estimated number of participants needed to achieve study objectives and how it was determined, including clinical and statistical assumptions supporting any sample size calculations	page 5, sample size calculation
Recruitment	15	Strategies for achieving adequate participant enrolment to reach target sample size	none
**Methods: Assignment of interventions (for controlled trials)**			
Allocation:			
Sequence generation	16a	Method of generating the allocation sequence (eg, computer-generated random numbers), and list of any factors for stratification. To reduce predictability of a random sequence, details of any planned restriction (eg, blocking) should be provided in a separate document that is unavailable to those who enrol participants or assign interventions	______________
Allocation concealment mechanism	16b	Mechanism of implementing the allocation sequence (eg, central telephone; sequentially numbered, opaque, sealed envelopes), describing any steps to conceal the sequence until interventions are assigned	______________
Implementation	16c	Who will generate the allocation sequence, who will enrol participants, and who will assign participants to interventions	page 2, study population
Blinding (masking)	17a	Who will be blinded after assignment to interventions (eg, trial participants, care providers, outcome assessors, data analysts), and how	open label, title
	17b	If blinded, circumstances under which unblinding is permissible, and procedure for revealing a participant's allocated intervention during the trial	not applicable

Methods: Data collection, management, and analysis

Data collection methods	18a	Plans for assessment and collection of outcome, baseline, and other trial data, including any related processes to promote data quality (eg, duplicate measurements, training of assessors) and a description of study instruments (eg, questionnaires, laboratory tests) along with their reliability and validity, if known. Reference to where data collection forms can be found, if not in the protocol	page 3/4; defining period, missing data
	18b	Plans to promote participant retention and complete follow-up, including list of any outcome data to be collected for participants who discontinue or deviate from intervention protocols	page 4, missing data
Data management	19	Plans for data entry, coding, security, and storage, including any related processes to promote data quality (eg, double data entry; range checks for data values). Reference to where details of data management procedures can be found, if not in the protocol	according to the guidelines of the EKNZ, not specially mentioned
Statistical methods	20a	Statistical methods for analysing primary and secondary outcomes. Reference to where other details of the statistical analysis plan can be found, if not in the protocol	page 5, statistical analysis
	20b	Methods for any additional analyses (eg, subgroup and adjusted analyses)	page 5, statistical analysis
	20c	Definition of analysis population relating to protocol non-adherence (eg, as randomised analysis), and any statistical methods to handle missing data (eg, multiple imputation)	page 5, statistical analysis

Methods: Monitoring

Data monitoring	21a	Composition of data monitoring committee (DMC); summary of its role and reporting structure; statement of whether it is independent from the sponsor and competing interests; and reference to where further details about its charter can be found, if not in the protocol. Alternatively, an explanation of why a DMC is not needed	page 5 ethical considerations, EKNZ is responsible
	21b	Description of any interim analyses and stopping guidelines, including who will have access to these interim results and make the final decision to terminate the trial	page 5 ethical considerations, EKNZ is responsible

Harms	22	Plans for collecting, assessing, reporting, and managing solicited and spontaneously reported adverse events and other unintended effects of trial interventions or trial conduct	page 5 ethical considerations, EKNZ is responsible
Auditing	23	Frequency and procedures for auditing trial conduct, if any, and whether the process will be independent from investigators and the sponsor	page 5 ethical considerations, EKNZ is responsible
**Ethics and dissemination**			
Research ethics approval	24	Plans for seeking research ethics committee/institutional review board (REC/IRB) approval	already registered
Protocol amendments	25	Plans for communicating important protocol modifications (eg, changes to eligibility criteria, outcomes, analyses) to relevant parties (eg, investigators, REC/IRBs, trial participants, trial registries, journals, regulators)	page 5 ethical considerations, EKNZ is responsible
Consent or assent	26a	Who will obtain informed consent or assent from potential trial participants or authorised surrogates, and how (see Item 32)	page 2, study population
	26b	Additional consent provisions for collection and use of participant data and biological specimens in ancillary studies, if applicable	not applicable
Confidentiality	27	How personal information about potential and enrolled participants will be collected, shared, and maintained in order to protect confidentiality before, during, and after the trial	page 2, study population
Declaration of interests	28	Financial and other competing interests for principal investigators for the overall trial and each study site	page 6
Access to data	29	Statement of who will have access to the final trial dataset, and disclosure of contractual agreements that limit such access for investigators	page 5 ethical considerations, EKNZ is responsible

Ancillary and post-trial care	30	Provisions, if any, for ancillary and post-trial care, and for compensation to those who suffer harm from trial participation	page 5 ethical considerations, EKNZ is responsible
Dissemination policy	31a	Plans for investigators and sponsor to communicate trial results to participants, healthcare professionals, the public, and other relevant groups (eg, via publication, reporting in results databases, or other data sharing arrangements), including any publication restrictions	none
	31b	Authorship eligibility guidelines and any intended use of professional writers	page 6
	31c	Plans, if any, for granting public access to the full protocol, participant-level dataset, and statistical code	page 6
**Appendices**			
Informed consent materials	32	Model consent form and other related documentation given to participants and authorised surrogates	on request available
Biological specimens	33	Plans for collection, laboratory evaluation, and storage of biological specimens for genetic or molecular analysis in the current trial and for future use in ancillary studies, if applicable	not applicable

*It is strongly recommended that this checklist be read in conjunction with the SPIRIT 2013 Explanation & Elaboration for important clarification on the items. Amendments to the protocol should be tracked and dated. The SPIRIT checklist is copyrighted by the SPIRIT Group under the Creative Commons "Attribution-NonCommercial-NoDerivs 3.0 Unported" license.
