# Supplementary material for: Are Routine Radiographs Needed the Day After Open Reduction and Internal Fixation Surgery for Distal Radius and Ankle Fractures: Study Protocol for a Prospective, Open Label, Randomized Controlled Trial
Source: JMIR Res Protoc. 2017 Aug 16;6(8):e159. doi: 10.2196/resprot.7698 (PMC5577453; doi:10.2196/resprot.7698)
Supplement: Multimedia Appendix 2 [file resprot_v6i8e159_app2.pdf]

Multimedia Appendix 1. Time schedule of enrollment and assessment for patients participating in this trial.

Figure A: Time schedule of enrolment, and assessments for patients participating in this trial.

	STUDY PERIOD				
	Enrolment	Allocation	X-Ray		
**TIMEPOINT**	**ER**	**Operation**	**Intraop**	**Postop**	**Week 6**
**ENROLMENT:**					
**Inclusion Criteria**	X				
**Informed consent**	X				
**Randomization**	X				
**Patients Baseline Characteristics**	X				
**INTERVENTIONS:**					
**X-Ray radius**					
Group 1	X	X	X	X	X
Group 2	X	X	X		X
**X-Ray OSG**					
Group 1	X	X	X	X	X
Group 2	X	X	X		X
**ASSESSMENTS:**					
**SF-36**	X	X			X
**Visual analog scale**					X
**Function scores (PRWHE or FAOS)**					X
**Range of motion**					X

Abbreviations: ER - Emergency Room; OSG - Ankle fracture group; SF-35 - Short Form 36 items; PRWHE: Patient Rated Wrist Hand Evaluation; FAOS: Foot and Ankle Outcome Score
